# Epidemiology of soy exposures and breast cancer risk

**DOI:** 10.1038/sj.bjc.6604145

**Published:** 2008-01-08

**Authors:** A H Wu, M C Yu, C-C Tseng, M C Pike

**Affiliations:** 1 Department of Preventive Medicine, Keck School of Medicine, University of Southern California, Los Angeles, CA, USA; 2 The Cancer Center, University of Minnesota, Minneapolis, MN, USA

**Keywords:** soy, Asian and Western populations, breast cancer

## Abstract

Most of the early studies published on soy and breast cancer were not designed to test the effect of soy; the assessment of soy intake was usually crude and few potential confounders were considered in the analysis. In this review, we focused on studies with relatively complete assessment of dietary soy exposure in the targeted populations and appropriate consideration for potential confounders in the statistical analysis of study data. Meta-analysis of the 8 (1 cohort, 7 case–control) studies conducted in high-soy-consuming Asians show a significant trend of decreasing risk with increasing soy food intake. Compared to the lowest level of soy food intake (⩽5 mg isoflavones per day), risk was intermediate (OR=0.88, 95% confidence interval (CI)=0.78–0.98) among those with modest (∼10 mg isoflavones per day) intake and lowest (OR=0.71, 95% CI=0.60–0.85) among those with high intake (⩾20 mg isoflavones per day). In contrast, soy intake was unrelated to breast cancer risk in studies conducted in the 11 low-soy-consuming Western populations whose average highest and lowest soy isoflavone intake levels were around 0.8 and 0.15 mg per day, respectively. Thus, the evidence to date, based largely on case–control studies, suggest that soy food intake in the amount consumed in Asian populations may have protective effects against breast cancer.

Soybeans and its products have been a staple in the Asian diet for centuries. Soybeans are the predominant source of isoflavones, one of the three main classes of phytoestrogens or plant oestrogens. Genistein and daidzein are the two major isoflavones; glycitein is a minor component. These soy isoflavone compounds are structurally similar to 17-*β* estradiol but possess weaker oestrogenic potencies. Given the recognised aetiologic association between oestrogen and breast cancer risk, there is biological plausibility that dietary soy intake may have anti-carcinogenic effect on the breast. A favoured mechanism by which soy isoflavones may influence breast cancer development is via their affinity and competition with endogenous oestrogens and other substrates in binding with oestrogen receptors (ERs). It has also been suggested that soy isoflavones may influence breast cancer risk via their anti-proliferative, anti-angiogenic, anti-oxidative and anti-inflammatory properties (Gilani and Anderson, 2002).

Lee *et al* (1991) first reported a reduced risk of breast cancer in premenopausal Singapore Chinese women who were high consumers of soy. Since then, there has been tremendous interest in the possible role of soy in the prevention of breast cancer. However, there is also the concern that soy may have stimulatory effects on the breast. Dietary genistein and highly processed soy agents have been found to stimulate growth in the MCF-7 breast cancer cells, and to promote breast cancer in ovaraectomised athymic mice models (for a review, see Messina *et al*, 2006). In a short term soy-feeding trial, women randomised to the soy arm exhibited significantly higher indices of increased DNA synthesis relative to women in the control arm, although this apparent association failed to persist following the inclusion of 33 additional subjects (Hargreaves *et al*, 1999).

The questions we would like to address in this review are as follows: (1) What is the epidemiologic evidence on soy and breast cancer? Is there a dose–response relationship? (2) Does the endogenous oestrogen environment, as reflected by menopausal status and body weight, affect this association? (3) Does timing of soy exposure affect this association? (4) Does the type of soy ingested affect this association, given that it can be consumed in the form of soy products that are common in a typical Asian diet or as fillers/extenders in processed foods?

We identified a total of 28 epidemiologic studies in English language with sufficiently detailed information on soy and breast cancer risk. Fourteen studies were included in the meta-analysis by Trock *et al* (2006). Of the remaining 14 studies, 6 were conducted in Asia (Hirose *et al*, 2005; Lee *et al*, 2005; Shannon *et al*, 2005; Ho *et al*, 2006; Do *et al*, 2007; Nishio *et al*, 2007) while the rest were conducted in various Western populations (Peterson *et al*, 2003; dos Santos Silva *et al*, 2004; Keinan-Boker *et al*, 2004; Bosetti *et al*, 2005; Thanos *et al*, 2006; Touillaud *et al*, 2006; Fink *et al*, 2007; Verheus *et al*, 2007). Given that Asians differ from Western populations in terms of types and amounts of soy consumed, and possess distinct characteristics linked to breast cancer risk, we conducted separate meta-analysis on epidemiologic data derived from Asians and Western populations, respectively.

## RISK ASSOCIATION STUDIES IN HIGH-SOY-CONSUMING ASIANS

In Asia, soy food is consumed traditionally as nonfermented soy foods (tofu, sometimes known as bean curd, soybeans and soy milk), fermented soy foods (miso and natto) or other soy products (fried, dried and pressed soy products), and the average intake is between 25 and 50 mg of isoflavones per day (Messina *et al*, 2006). In contrast, intake of soy isoflavones in Western populations is usually less than 1.0 mg of isoflavones per day and Asian soy foods are rarely consumed. Their sources of soy isoflavones come from some legumes, sprouts and vegetables containing small amounts of isoflavones, and from soy flour and soy protein that are commonly added as extenders and fillers in different bakery and canned goods (Horn-Ross *et al*, 2001).

Eight of the 14 studies conducted in Asia or in Asian Americans captured the main sources of soy intake and carefully adjusted for relevant dietary and non-dietary potential confounders. We excluded the remaining six (two cohort, four case–control) studies from the meta-analysis due to incomplete assessment of soy intake in those studies. Specifically, two studies (Wu *et al*, 1996; Ho *et al*, 2006) examined only about intake of tofu. Four other studies (Yuan *et al*, 1995; Key *et al*, 1999; Hirose *et al*, 2003; Nishio *et al*, 2007) asked about intake of several soy foods that captured between 40 and 60% of total soy intake in those populations.

In the eight (one cohort, seven case–control) studies with relatively complete assessment of total soy intake, dietary soy exposure was expressed in varying units, including grams of soy foods per day (Lee *et al*, 1991, 2005; Hirose *et al*, 2005; Shannon *et al*, 2005; Do *et al*, 2007), grams of soy protein per day (Lee *et al*, 1991; Dai *et al*, 2001), and milligrams of soy isoflavones per day (Wu *et al*, 2002; Yamamoto *et al*, 2003; Hirose *et al*, 2005). To facilitate comparison between individual studies, all dietary soy exposures were converted to units of milligram of isoflavones assuming that 1 g of soy protein or 10 g of soy food contain 3 mg of isoflavones (Messina *et al*, 2006; Trock *et al*, 2006).

**Figure 1:**
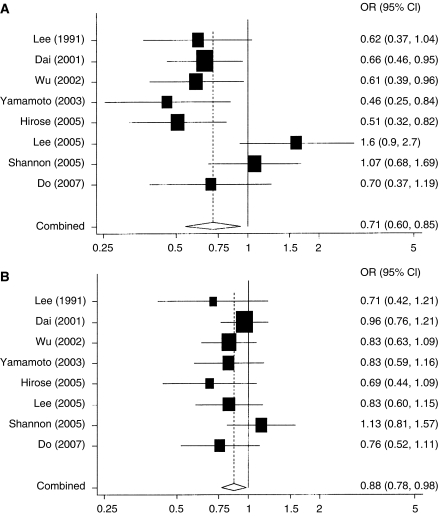
(**A**) Highest (∼20 mg or more isoflavones per day) *vs* lowest (∼5 mg or less isoflavone per day) level of soy intake and breast cancer – eight studies conducted in Asia and in Asian Americans. (**B**) Moderate (∼10 mg isoflavones per day) *vs* lowest (∼5 mg or less isoflavone per day) level of soy intake and breast cancer – eight studies conducted in Asia and in Asian Americans. See footnote e of Table 1.

Table 1 shows the meta-analysis of these eight studies with information on total soy food intake and breast cancer in Asian women. There was a statistically significant 29% reduction in the risk of breast cancer associated with high soy intake (summary OR=0.71, 95% confidence interval (CI)=0.60–0.85) compared to the lowest level of soy intake (Table 1). Results of the individual studies are shown in Figure 1A. Across these Asian studies, the median cut point of the highest intake of soy isoflavones was 20 mg or more per day and the lowest intake was 5 mg or less per day. A moderate level of soy intake (median was approximately 10 mg isoflavones per day) was associated with a statistically significant 12% reduction in risk (summary OR=0.88, 95% CI=0.78–0.98) compared to the lowest level of soy intake (Figure 1B). All but one of the studies was conducted in Asia; exclusion of the study conducted among Asian Americans (Wu *et al*, 2002) did not materially change the overall summary risk estimate. Only one of these studies was a cohort study (Yamamoto *et al*, 2003); the summary OR of the seven case–control studies combined was similar to overall results. Test of heterogeneity across these eight studies comparing highest *vs* lowest level of soy intake showed statistically significant heterogeneity (*P*=0.023) that was due largely to the findings of one hospital-based case–control study, which found an increased risk associated with high soy intake (Lee *et al*, 2005) (Figure 1A). The results across the remaining seven studies were comparable; the summary OR was 0.66 (95% CI=0.55–0.78) (*P* heterogeneity=0.32) comparing the highest to the lowest level of soy intake. There was no significant heterogeneity across these eight studies comparing moderate *vs* lowest level of soy intake (*P* heterogeneity=0.60) (Figure 1B).

Results were presented by menopausal status in six of these studies; a significant inverse association was observed in both pre-menopausal (summary OR=0.65, 95% CI=0.50–0.85) and postmenopausal women (summary OR=0.63, 95% CI=0.46–0.85) (Table 1). Few studies have investigated whether body size modify the soy–breast cancer association, particularly in postmenopausal women. Investigators in Shanghai, China, reported that the inverse soy–breast association was more evident in women with higher body mass index (BMI) (⩾25 kg m^−2^) than their lower BMI counterparts (Dai *et al*, 2001). The authors did not perform separate analyses in the pre- *vs* postmenopausal women whose endogenous oestrogen environments were vastly different. Premenopausal women represented about 65% of the subjects in this Shanghai study. In our Los Angeles Asian Breast Cancer study, we did not observe any clear modifying effects of BMI on the soy–breast cancer association in either pre- or postmenopausal women (unpublished data).

Two studies asked about intake of soy during adolescence. In Shanghai, soy intake during adolescence is associated with a greater risk reduction than adult intake (Shu *et al*, 2001). Similarly, in the Los Angeles Asian Breast Cancer Study, we found that soy intake during adolescence exhibited a stronger protective effect on risk than adulthood exposure (Wu *et al*, 2002). Compared to women who were low soy consumers during both adolescence and adult life, women who were low soy consumers during adolescence but were high-soy consumers during adult life displayed modestly reduced risk without reaching statistical significance (0.88, 95% CI=0.61–1.18) (Wu *et al*, 2007a).

We also examined the results of the six excluded studies (due to incomplete soy assessment) (Yuan *et al*, 1995; Wu *et al*, 1996; Key *et al*, 1999; Hirose *et al*, 2003; Ho *et al*, 2006; Nishio *et al*, 2007) with findings of our meta analysis based on the remaining eight studies in Asians (Table 1). A comparison of the reported ORs and their 95% CIs in the excluded studies with the summary OR (95% CI) showed the results of the excluded studies to be mostly compatible with the findings of the meta-analysis.

## RISK ASSOCIATION STUDIES IN LOW-SOY- CONSUMING WESTERN POPULATIONS

In studies conducted in Western populations (including one study of South Asians in the United Kingdom, daily soy isoflavone intake was estimated based on intake of certain traditional Asian soy foods and other food items for which values of isoflavones are available and have been compiled into various databases. Average intake of soy isoflavones was low (median was approximately 0.3 mg day^−1^) in these Western populations. Traditional Asian soy foods contributed little to the total intake of soy isoflavones in these studies: it was 3% in Norfolk-United Kingdom (Grace *et al*, 2004), 7% in Ultrecht, the Netherlands (Keinan-Boker *et al*, 2004), 24% in Germany (Linseisen *et al*, 2004) and 35% in the San Francisco Bay Area, USA (Horn-Ross *et al*, 2001). Instead a considerable (25–60%) proportion of soy isoflavones in these populations was from soy components added to typical Western foods including bakery and canned products.

**Figure 2:**
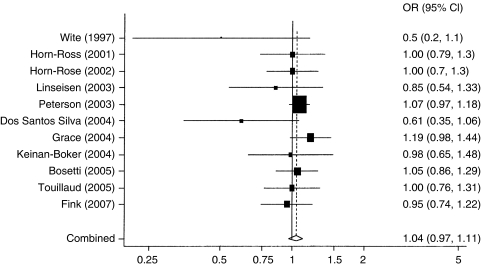
Highest (∼0.8 mg or more isoflavones per day) *vs* lowest (∼0.15 mg or less isoflavones per day) level of soy intake and breast cancer – 11 studies conducted in Western populations. Odds ratio (OR), 95% CI.

Table 2 presents the meta-analysis results of these 11 studies in Western populations, showing no association between adult soy intake and breast cancer risk; the summary OR was 1.04 (95% CI=0.97–1.11) between the highest (median was 0.8 mg or more per day) *vs* lowest (median was 0.15 mg or less per day) soy isoflavone intake. Results of the individual studies are shown in Figure 2. There was no significant heterogeneity among the study results (*P*=0.42). The summary ORs and corresponding 95% CIs obtained from the four cohort/nested case–control studies and the seven case–control studies are overlapping.

In a few of these studies conducted in Western populations (Witte *et al*, 1997; Horn-Ross *et al*, 2001), risk patterns in relation to Asian soy foods (i.e., tofu and others) were presented. Results on these soy foods, at the relatively low levels consumed in the West, are compatible with those at corresponding consumption levels in Asian women.

To overcome the difficulties and imprecision associated with estimating low dietary soy intake from questionnaires, baseline, prediagnostic blood/urine isoflavone levels collected from participants of three cohorts were examined in relation to breast cancer risk (den Tonkelaar *et al*, 2001; Grace *et al*, 2004; Verheus *et al*, 2007); two of these studies also had information on dietary soy isoflavone intake (Grace *et al*, 2004; Keinan-Boker *et al*, 2004). The combined OR (summary OR=1.02, 95% CI=0.84–1.23) from these three nested case–control studies is consistent with the finding of no association based on dietary soy isoflavones and breast cancer risk in Western populations. There were no differences in results by menopausal status (Horn-Ross *et al*, 2001; dos Santos Silva *et al*, 2004; Keinan-Boker *et al*, 2004; Bosetti *et al*, 2005; Fink *et al*, 2007; Verheus *et al*, 2007). One study examined the soy–breast cancer association by body size and reported no differences in results (Bosetti *et al*, 2005).

Soy intake during adolescence and breast cancer risk was investigated in one large Canadian case–control study (Thanos *et al*, 2006). The authors reported a significant, inverse association with a score representing intake of foods considered rich in isoflavones. In this study population, few subjects (<5%) reported intake of any soy foods (tofu, soybeans, soy milk and soy powder drinks) and the most likely sources of soy isoflavones were pancakes (mix), canned tuna and processed meats. The authors did not perform separate analyses by type of soy consumed (as food additives *vs* as soy foods). Due to the possible differential effects of the two sources of dietary soy exposures on breast cancer risk, it is unclear if these findings truly reflect a beneficial effect of early life soy intake on breast cancer development.

## SOY AND BREAST CANCER SURVIVAL

Few studies have investigated the effects of soy intake in women with breast cancer. Among Chinese women in Shanghai, usual soy food intake before breast cancer diagnosis was unrelated to disease-free breast cancer survival after a median follow-up of 5.2 years, but information on tamoxifen use was not included (Boyapati *et al*, 2005). To address the concern that soy may negate the effect of tamoxifen (Messina *et al*, 2006), we investigated and found no relationship between serum levels of tamoxifen or its metabolites and self-reported intake of soy or serum levels of isoflavones among a population-based sample of Asian-American women with breast cancer (Wu *et al*, 2007b). Further studies are needed to determine the effects of soy (before and after breast cancer diagnosis), in relation to tamoxifen use and breast cancer outcome.

## SOY AND MARKERS OF BREAST CANCER RISK

It has been speculated that the protective effect of soy intake on breast cancer could be mediated through a hormonal mechanism, and that high soy intake may affect circulating levels of oestrogen and sex-hormone-binding globulins and mammographic densities in a favourable manner (Messina *et al*, 2006). There is suggestion in some cross-sectional studies that circulating oestrogen and androgen levels may be nonsignificantly decreased in association with high-soy food intake but these effects are typically small and the results are not consistent. Results from the nearly 20 short-term intervention studies on soy (mainly given as supplements or other soy protein products) and circulating oestrogen and sex-hormone-binding globulin are mixed and this may be related to differences in study design, small sample sizes, the amount and source of soy isoflavones that were used, duration of supplementation and other reasons (for a review, see Maskarinec *et al*, 2004; Wu *et al*, 2005).

Fewer studies have investigated the effect of soy intake on mammographic density. The strongest evidence that mammographic density is significantly reduced in association with high-soy food intake is based on a cross-sectional study we conducted among Chinese women in Singapore. In another cross-sectional study conducted in Hawaii, high soy intake was associated with lower mammographic density among Chinese and Japanese women in Hawaii but higher mammographic density in Caucasians and Hawaiians; none of the associations was statistically significant. Three intervention studies of 1–2 years duration, conducted in mostly premenopausal or perimenopausal women, failed to note any effects of soy, given as soy foods, red clover isoflavones or isoflavone supplements, on mammographic density (for a review, see Messina *et al*, 2006; Ursin *et al*, 2006).

Thus, the collective evidence based on circulating hormones and mammographic density does not show any strong or consistent oestrogenic or anti-oestrogenic effects of soy on the breast. Differences in intestinal microflora can influence the subsequent absorption and metabolism of soy intake. Approximately one-third of the population is capable of producing equol, a metabolite of daidzein. It has been suggested that differences in a woman's ability to produce equol may influence the extent to which her soy consumption can impact on her risk of breast cancer development (Setchell *et al*, 2002). At present, it is unclear whether failure to take account of the differences in soy metabolism between individuals has contributed to the inconsistencies between studies.

## CONCLUSIONS

Overall data based on Asian women, mainly derived from case–control studies, show a dose-dependent, statistically significant association between soy food intake and breast cancer risk reduction. There was an approximately 16% risk reduction per 10 mg of isoflavones intake per day. Age at exposure may be a co-determinant of risk; adolescent intake shows a stronger effect on risk than intake during adulthood. Soy intake was unrelated to breast cancer risk in studies conducted in Western populations in which the average intake of soy isoflavones was low (<1 mg day^−1^) and exposure is mainly in the form of soy components added as fillers/extenders to typical Western foods. There is little evidence of a modifying effect by menopausal status or body weight on the soy–breast cancer association. It is inconclusive whether habitual soy intake or short-term soy supplementation influences circulating hormone levels or mammographic density. If the beneficial effect of Asian soy foods on breast cancer risk noted above is real, their mechanisms of action remain to be elucidated.

## Figures and Tables

**Table 1:** Soy intake and breast cancer risk – eight studies conducted in Asia and in Asian Americans

**Description**	**No. of studies**	**Odds ratio**	**95% confidence interval**
*Highest (∼20 mg or more isoflavone per day) vs lowest (∼5 mg or less isoflavone per day)*			
All studies^a^	8	0.71	(0.60–0.85)
All studies in Asia^b^	7	0.73	(0.61–0.89)
Case–control studies^c^	7	0.75	(0.62–0.89)
Premenopausal women^d^	6	0.65	(0.50–0.85)
Postmenopausal women^d^	6	0.63	(0.46–0.85)
			
*Moderate (∼10 mg isoflavone per day) vs lowest (∼5 mg isoflavone or less per day)*			
All studies^e^	8	0.88	(0.78–0.98)

a Studies included in meta-analysis: Lee *et al* (1991); Dai *et al* (2001); Wu *et al* (2002); Yamamoto *et al* (2003); Hirose *et al* (2005); Lee *et al* (2005); Shannon *et al* (2005); Do *et al* (2007). We calculated ORs associated with soy products (in g) for pre- and postmenopausal women combined in the study by Lee *et al* (1991); the results in postmenopausal women in this study were published in a 1992 paper. We also calculated ORs for pre- and postmenopausal women combined in Hirose *et al* (2005).

b Excluded Wu *et al* (2002) in the analysis.

c Excluded Yamamoto *et al* (2003) in the analysis.

d Results were not presented by menopausal status in two studies (Dai *et al*, 2001; Shannon *et al* 2005) and they were excluded in this analysis. Do *et al* (2007) published the data in pre- and postmenopausal women separately in a subsequent paper in 2007.

e Results were presented by tertiles (Lee *et al*, 1991; Hirose *et al*, 2005), quartiles (Wu *et al*, 2002; Yamamoto *et al*, 2003; Lee *et al*, 2005; Shannon *et al*, 2005; Do *et al*, 2007) and deciles (Dai *et al*, 2001) of soy intake. Risk estimate for moderate intake was calculated using the ORs for deciles 5 and 6 in Dai *et al* (2001) and the ORs for quartiles 2 and 3 in studies which presented results by quartiles of soy intake.

**Table 2:** Soy intake and breast cancer risk – 11 studies conducted in Western populations

**Description**	**No. of studies**	**Odds ratio**	**95% confidence interval**
*Highest (∼0.8 mg or more isoflavone per day) vs lowest (∼0.15 mg or less isoflavone per day)*			
All^a^	11	1.04	(0.97–1.11)
Cohort/nested case–control^b^	4	1.08	(0.95–1.24)
Case–control studies^c^	7	1.02	(0.95–1.11)

a Studies included in meta-analysis: Witte *et al* (1997); Horn-Ross *et al* (2001, 2002); Linseisen *et al* (2003); Peterson *et al* (2003); dos Santos Silva *et al* (2004); Grace *et al* (2004); Keinan-Boker *et al* (2004); Bosetti *et al* (2005); Touillaud *et al* (2006); Fink *et al* (2007). We estimated daily soy isoflavone intake in one study (Witte *et al* (1997) by assuming that one serving of tofu per week is 50 g and this is equivalent to 2.14 mg isoflavone per day.

b Four cohort/nested case–control studies: Horn-Ross *et al* (2002); Grace *et al* (2004); Keinan-Boker *et al* (2004); Touillard *et al* (2006).

c Seven case–control studies: Witte *et al* (1997); Horn-Ross *et al* (2001); Linseisan *et al* (2003); Peterson *et al* (2003); dos Santos Silva *et al* (2004); Bosetti *et al* (2005); Fink *et al* (2007).
